# Development and validation of nomograms for predicting survival outcomes in patients with T1-2N1 breast cancer to identify those who could not benefit from postmastectomy radiotherapy

**DOI:** 10.3389/fonc.2023.1112687

**Published:** 2023-03-28

**Authors:** Hongyu Pu, Yunbo Luo, Linxing Zhang, Xin Li, Fangwei Li, Jingtai Chen, Shuangqiang Qian, Yunhui Tang, Xiaobo Zhao, Lingmi Hou, Yanchun Gao

**Affiliations:** ^1^ Department of Breast and Thyroid Surgery, Affiliated Hospital of North Sichuan Medical College, Nanchong, China; ^2^ Department of Hepatobiliary Surgery I, Affiliated Hospital of North Sichuan Medical College, Nanchong, China; ^3^ Department of Breast and Thyroid Surgery, Chongqing General Hospital, Chongqing, China; ^4^ Department of Breast and Thyroid Surgery, Guang’an People’s Hospital, Chongqing, China

**Keywords:** T1-2N1 breast cancer, postmastectomy radiotherapy, nomogram, overall survival, breast cancer-specific survival

## Abstract

**Purpose:**

In this study, we aimed to develop and validate nomograms for predicting the survival outcomes in patients with T1-2N1 breast cancer to identify the patients who could not benefit from postmastectomy radiotherapy (PMRT).

**Methods:**

Data from 10191 patients with T1-2N1 breast cancer were extracted from the Surveillance, Epidemiology, and End Results (SEER) database. Of them, 6542 patients who had not received PMRT formed the training set. Concurrently, we retrospectively enrolled 419 patients from the Affiliated Hospital of North Sichuan Medical College (NSMC), and 286 patients who did not undergo PMRT formed the external validation set. The least absolute shrinkage and selection operator (LASSO) and multivariate Cox regression analyses were used for selecting prognostic factors in the training set. Using the selected factors, two prognostic nomograms were constructed. The nomograms’ performance was assessed using the concordance index (C-index), calibration curves, decision curve analysis (DCA), and risk subgroup classification. The stabilized inverse probability of treatment weights (IPTWs) was used to balance the baseline characteristics of the different risk groups. Finally, the survival outcomes and effectiveness of PMRT after IPTW adjustment were evaluated using adjusted Kaplan–Meier curves and Cox regression models.

**Results:**

The 8-year overall survival (OS) and breast cancer-specific survival (BCSS) rates for the SEER cohort were 84.3% and 90.1%, with a median follow-up time of 76 months, while those for the NSMC cohort were 84.1% and 86.9%, with a median follow-up time of 73 months. Moreover, significant differences were observed in the survival curves for the different risk subgroups (P < 0.001) in both SEER and NSMC cohorts. The subgroup analysis after adjustment by IPTW revealed that PMRT was significantly associated with improved OS and BCSS in the intermediate- (hazard ratio [HR] = 0.72, 95% confidence interval [CI]: 0.59–0.88, P=0.001; HR = 0.77, 95% CI: 0.62–0.95, P = 0.015) and high- (HR=0.66, 95% CI: 0.52–0.83, P<0.001; HR=0.74, 95% CI: 0.56–0.99, P=0.039) risk groups. However, PMRT had no significant effects on patients in the low-risk groups.

**Conclusion:**

According to the prognostic nomogram, we performed risk subgroup classification and found that patients in the low-risk group did not benefit from PMRT.

## 1 Introduction

In 2016, the American Society of Clinical Oncology updated the joint guidelines regarding the use of PMRT in patients with T1-2N1 breast cancer (1). The panel emphasized that the available evidence indicates that postmastectomy radiotherapy (PMRT) reduces the risk of recurrence and mortality. However, in certain subsets of patients with T1-2N1 breast cancer, the risk of recurrence is so low that PMRT offers a limited survival advantage. Therefore, PMRT should not be routinely recommended for such patients. At the 2017 St. Gallen International Expert Consensus Conference, the panel proposed the consideration of PMRT omission in patients with 1 to 3 positive lymph nodes with favorable biological and histological characteristics (2).

Accumulated evidence has confirmed that the effectiveness of PMRT is heterogeneous among patients with T1-2N1 breast cancer (3). Therefore, selecting which patients with 1 to 3 positive lymph nodes are suitable for radiotherapy is a current issue being explored by many scholars (4–7). Such a problem has also attracted widespread attention in the field of breast cancer, with many studies attempting to find appropriate methods to solve the above problem. Unfortunately, a consensus has not been reached regarding the criteria for exempting PMRT (8).

The advantage of the nomogram is its ability to incorporate all independent prognostic predictors to personalize survival prediction for more accurate clinical decision-making. In this study, we sought to develop nomograms for predicting the overall survival (OS) and breast cancer-specific survival (BCSS), and performed risk stratification to distinguish the subgroup of patients with T1-2N1 breast cancer that might not benefit from PMRT. Additionally, the nomograms we constructed were externally validated using a single cohort of 419 patients from a multi-center registered clinical trial (NCT00041119).

## 2 Materials and methods

### 2.1 Study design and cohort selection

**Figure 1:**
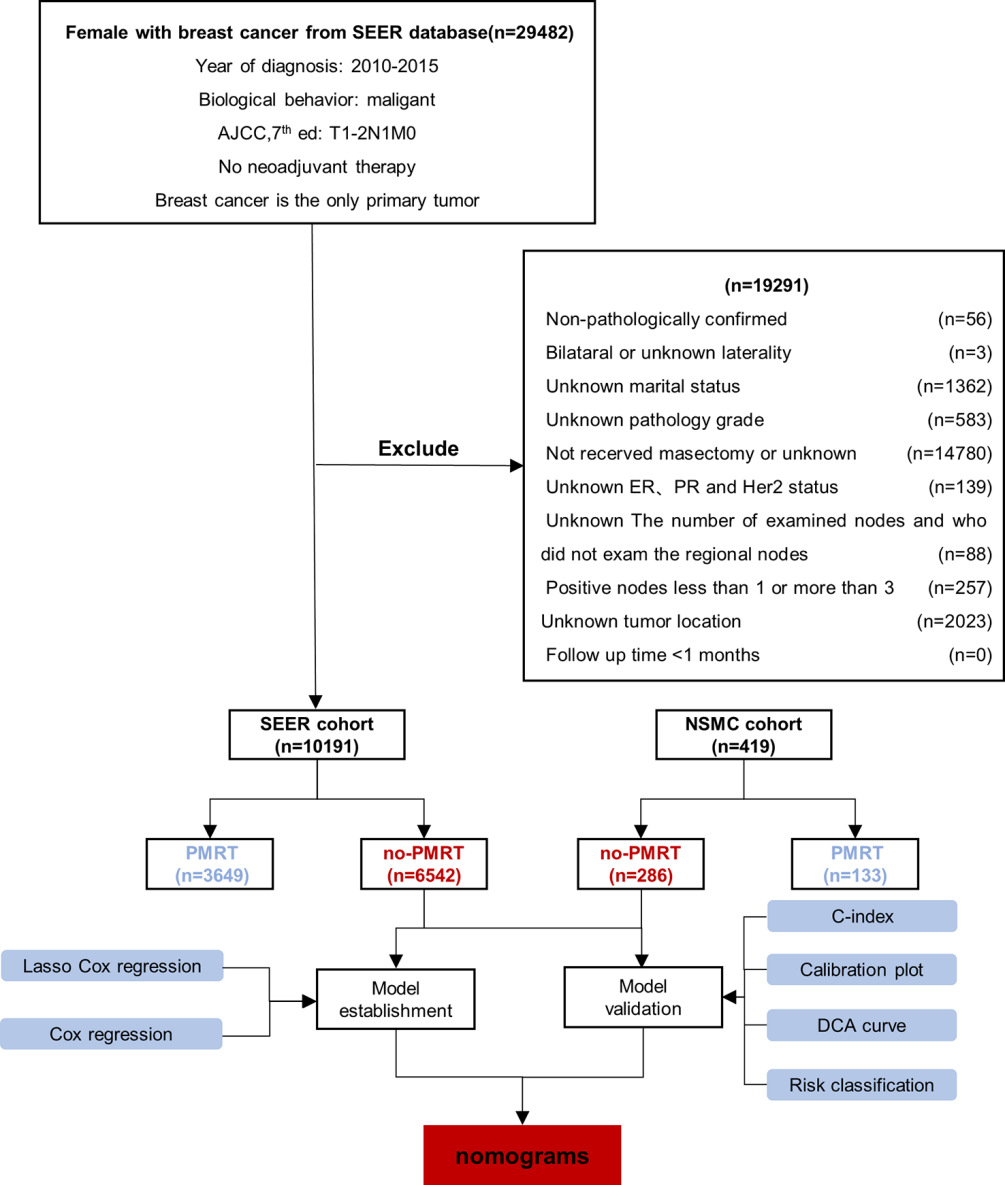
Patients’ enrollment and exclusion flow chart. The SEER cohort comprised 10191 patients, which were further separated into the no-PMRT (training set, n=6542) and PMRT group (n=3649). The NSMC cohort consisted of 419 patients, who were further divided into the no-PMRT (n=286) and PMRT group (n=133). PMRT, postmastectomy radiotherapy.

Data regarding 10191 patients with T1-2N1 breast cancer, diagnosed between 2010 and 2015, were extracted from the Surveillance, Epidemiology, and End Results (SEER) database, designated the SEER cohort, of which 6542 patients who did not receive PMRT were separated into a training set (**Figure 1**). The inclusion criteria were as follows: (1) female patients with breast cancer whose year of diagnosis was 2010-2015; (2) malignant disease as per the International Classification of Diseases for Oncology, 3rd edition, (3) T1-2N1M0 (American Joint Committee on Cancer [AJCC] 7th edition), 4) not receiving neoadjuvant therapy, and (5) breast cancer was the only primary malignancy. Patients with (1) no pathological confirmation; (2) bilateral or unspecified side; (3) unknown marital status; (4) unknown histological grade; (5) no mastectomy or unknown surgical procedure; (6) unknown estrogen receptor (ER), progesterone receptor (PR), or human epidermal growth factor receptor 2 (Her2) status; (7) unknown number of examined and number of positive lymph nodes other than 1 to 3; (8) unknown tumor location; and (9) survival time <1 month were excluded from the study.

Concurrently, we recruited a retrospective cohort of patients diagnosed with pT1-2N1M0 breast cancer between January 2010–December 2015 from the Affiliated Hospital of North Sichuan Medical College (NSMC). Female patients (1) with histologically diagnosed invasive breast cancer, (2) with unilateral cancer, (3) with 1–3 positive lymph nodes, (4) who did not receive neoadjuvant therapy, (5) who underwent mastectomy plus sentinel lymph node biopsy or axillary lymph node dissection, and (6) without multiple primary malignancies were included in the study. We included 419 patients, designated as the NSMC cohort, in the analysis. From the NSMC cohort, 286 patients who did not receive PMRT formed an external validation set (**Figure 1**).

### 2.2 Variable definitions and comments

The variables registered in this study included age at diagnosis, marital status, tumor laterality, tumor location, histological type, histological grade, T stage, number of positive lymph nodes, lymph node ratio (LNR), and ER, PR, Her2, and chemotherapy status.

Age at diagnosis was analyzed as a categorical variable (every ten years) except for ages less than 40 years and more than 70 years. Marital status was classified as married or other (unmarried/separated/divorced/widowed). Tumor laterality was classified as left or right. Tumor location was classified as inner + central or others (upper-outer/lower-outer/axillary tail/overlapping lesion). The histological type was classified as infiltrating duct carcinoma, infiltrating lobular carcinoma, or others. The histological grade was classified as I (well-differentiated), II (moderately differentiated), or III (poorly differentiated and undifferentiated). All patients were staged according to the AJCC 7th edition staging system. LNR was defined as the ratio of positive nodes to dissected nodes, reflecting the burden of axillary tumors and the extent of surgery. The ratio was categorized as ≤ 15% or > 15%. ER, PR, and Her2 status were classified as negative or positive. The chemotherapy status was classified as either no or yes.

### 2.3 Follow-up and outcomes

The study’s primary endpoints were OS and BCSS. OS was defined as the time from diagnosis to death or the last follow-up, and BCSS was defined as the time from diagnosis to death from breast cancer or the last follow-up.

### 2.4 Statistical analysis

Follow-up times were reported using the median and interquartile range (IQR), while categorical variables were described using frequencies and percentages. Groups were compared using the chi-square test or Fisher’s exact test. Time-to-event variables were evaluated using the Kaplan–Meier method, and groups were compared using the log-rank test.

The least absolute shrinkage and selection operator (LASSO) regression analysis was used to select potential predictive variables preliminarily. Backward stepwise selection with the Akaike information criterion was used to further identify variables for the multivariable Cox regression models. The hazard ratio (HR) was calculated with 95% confidence intervals (CIs). Finally, the selected variables were incorporated into the nomograms to predict the 3-year, 5-year, and 8-year OS and BCSS in patients with T1-2N1 breast cancer. Regression coefficients were applied to each observation to generate a linear predictor for assigning points to each patient in the nomograms. The performance of the two nomograms was evaluated in the training and validation sets. A concordance index (C-index) was used to evaluate the model’s discriminative ability. Calibration curves (bootstrap = 1000) were generated to estimate the prediction model’s consistency, while decision curve analysis (DCA) was used to assess the nomograms’ net benefits in the clinical context.

The optimal cut-off values for the total points of the nomograms were determined using X-tile software (version 3.6.1). All patients in the SEER database (10191) were reclassified into low-, intermediate-, and high-risk groups. To reduce the confounding bias, we performed stabilized inverse probability of treatment weights (IPTWs), a propensity score-based method used to balance baseline variables without sample loss (9). Survival analyses of the IPTW-adjusted cohort were performed using the adjusted Kaplan-Meier method and log-rank test. Adjusted HR and 95% CI were calculated to estimate the treatment effects of PMRT. All statistical analyses were performed using R software (version 4.2.1). All tests were two-sided, and P < 0.05 indicated a statistically significant difference.

## 3 Results

### 3.1 Baseline characteristics

Of the 10191 patients selected from the SEER database, 6542 (64.2%) patients who did not receive PMRT were included in the no-PMRT group. In the NSMC cohort, including 419 patients with pT1-2N1M0 breast cancer, 286 (68.3%) did not receive PMRT and were assigned as an external validation set. The detailed baseline characteristics of all patients are listed in **Table 1**. The proportion of patients who received PMRT was similar between the SEER and NSMC cohorts, and the observed associations were comparable. In the SEER cohort, compared with patients who did not receive PMRT, those who received PMRT were younger, had a significantly higher proportion patients with married, infiltrating duct carcinoma, grade III, T2 stage tumor, three positive lymph nodes, LNR >15%, ER-negative, PR-negative, and received chemotherapy. The PMRT group in the NSMC cohort had a significantly lower proportion of patients with grade I disease, T1 stage, one positive lymph node, LNR ≤ 15%, and not receiving chemotherapy.

**Table 1:** Baseline characteristics of the SEER and NSMC cohorts in T1-2N1 breast cancer.

Characteristic	SEER cohort (n=10191)			NSMC cohort (n=419)		
	no-PMRT	PMRT	P-value	no-PMRT	PMRT	P-value
	(n=6542)	(n=3649)		(n=286)	(n=133)	
**Age (years)**			<0.001			0.462
<40	483 (7.4)	479 (13.1)		24 (8.4)	13 (9.8)	
40-49	1516 (23.2)	983 (26.9)		58 (20.3)	32 (24.1)	
50-59	1783 (27.3)	979 (26.8)		79 (27.6)	38 (28.6)	
60-69	1478 (22.6)	757 (20.7)		65 (22.7)	32 (24.1)	
≥70	1282 (19.6)	451 (12.4)		60 (21.0)	18 (13.5)	
**Marital status**			0.002			0.241
Married	3981 (60.9)	2332 (63.9)		175 (61.2)	90 (67.7)	
Others	2561 (39.1)	1317 (36.1)		111 (38.8)	43 (32.3)	
**Tumor laterality**			0.352			0.757
Center	3295 (50.4)	1802 (49.4)		140 (49.0)	68 (51.1)	
Right	3247 (49.6)	1847 (50.6)		146 (51.0)	65 (48.9)	
**Tumor location**			0.524			0.782
Inner+central	1683 (25.7)	917 (25.1)		74 (25.9)	32 (24.1)	
Others	4859 (74.3)	2732 (74.9)		212 (74.1)	101 (75.9)	
**Histological type**			0.015			0.676
Infiltrating duct carcinoma	557 (8.5)	362 (9.9)		26 (9.1)	14 (10.5)	
Infiltrating lobular carcinoma	5050 (77.2)	2816 (77.2)		222 (77.6)	98 (73.7)	
Others	935 (14.3)	471 (12.9)		38 (13.3)	21 (15.8)	
**Grade**			<0.001			0.031
I	998 (15.3)	377 (10.3)		40 (14.0)	7 (5.3)	
II	3175 (48.5)	1688 (46.3)		143 (50.0)	73 (54.9)	
III	2369 (36.2)	1584 (43.4)		103 (36.0)	53 (39.8)	
**T stage**			<0.001			0.005
T1	2982 (45.6)	1280 (35.1)		140 (49.0)	45 (33.8)	
T2	3560 (54.4)	2369 (64.9)		146 (51.0)	88 (66.2)	
**Positive lymph nodes**			<0.001			0.036
1	4343 (66.4)	1682 (46.1)		178 (62.2)	67 (50.4)	
2	1586 (24.2)	1160 (31.8)		76 (26.6)	41 (30.8)	
3	613 (9.4)	807 (22.1)		32 (11.2)	25 (18.8)	
**LNR (%)**			<0.001			0.008
≤15	3552 (54.3)	1514 (41.5)		162 (56.6)	56 (42.1)	
>15	2990 (45.7)	2135 (58.5)		124 (43.4)	77 (57.9)	
**ER**			0.023			1.000
Negative	902 (13.8)	564 (15.5)		40 (14.0)	18 (13.5)	
Positive	5640 (86.2)	3085 (84.5)		246 (86.0)	115 (86.5)	
**PR**			0.001			0.897
Negative	1526 (23.3)	961 (26.3)		72 (25.2)	35 (26.3)	
Positive	5016 (76.7)	2688 (73.7)		214 (74.8)	98 (73.7)	
**Her2**			0.624			0.348
Negative	5485 (83.8)	3045 (83.4)		250 (87.4)	111 (83.5)	
Positive	1057 (16.2)	604 (16.6)		36 (12.6)	22 (16.5)	
**Chemotherapy**			<0.001			0.013
No	1894 (29.0)	535 (14.7)		86 (30.1)	24 (18.0)	
Yes	4648 (71.0)	3114 (85.3)		200 (69.9)	109 (82.0)	
Outcomes						
**median follow-up months (IQR)**	76 (58-97)			73 (58-95.5)		
**8-Y OS**	84.3 (83.5-85.2)			84.1 (80.1-88.4)		
**8-Y BCSS**	90.1 (89.5-90.8)			86.9 (82.9-91.1)		

SEER, surveillance, epidemiology, and end results database; NSMC, Affiliated Hospital of North Sichuan Medical College; PMRT, postmastectomy radiotherapy; ER, estrogen receptor; PR, progesterone receptor; Her2, Human epidermal growth factor receptor 2; LNR, lymph node ratio. IQR, interquartile range; OS, overall survival; BCSS, breast cancer-specific survival.

In the SEER cohort, the median follow-up time was 76 (IQR: 58–97) months, with an 8-year OS rate of 84.3% and an 8-year BCSS rate of 90.1%. In the NSMC cohort, the median follow-up time was 73 (IQR: 58–95.5) months, with an 8-year OS rate of 84.1% and an 8-year BCSS rate of 86.9%.

### 3.2 Identification of prognostic factors

**Figure 2:**
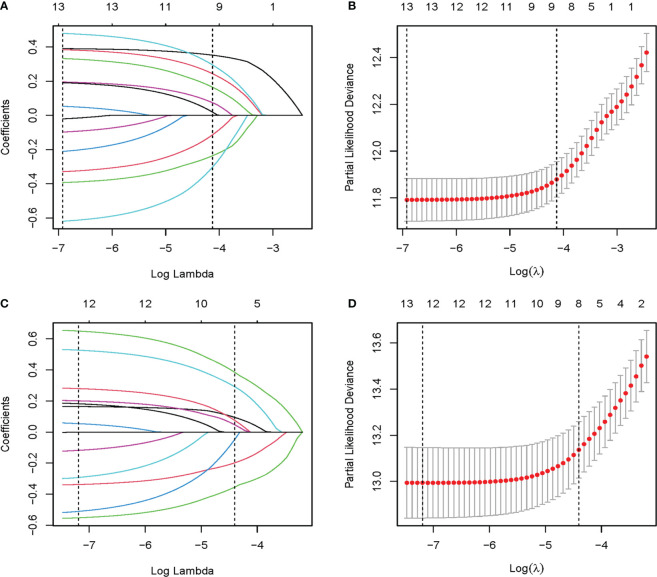
The LASSO regression used to select prognostic factors. **(A)** LASSO coefficient profiles of 13 variables for OS; **(B)** LASSO Cox analysis identified 9 variables for OS; **(C)** LASSO coefficient profiles of 13 variables for BCSS; **(D)** LASSO Cox analysis identified 8 variables for BCSS. OS, overall survival; BCSS, breast cancer-specific survival; LASSO, the least absolute shrinkage and selection operator.

Established risk factors, as well as demographic and tumor characteristics of clinical importance, were selected as candidate variables for the prediction model. Initially, 13 candidate variables were included in the analysis. Following the LASSO regression analysis, age, marital status, grade, T stage, number of positive lymph nodes, LNR, and ER, PR, and chemotherapy status were identified as prognostic factors associated with OS. The factors associated with BCSS included age, marital status, grade, T stage, number of positive lymph nodes, and ER, PR, and Her2 status (**Figure 2**).

Multivariate analysis of these factors found that age < 40 years, married status, grade I, T1, one positive lymph node, LNR ≤ 15%, ER positivity, PR positivity, and chemotherapy were independent factors associated with better OS. Similarly, age < 40 years, married status, grade I, T1, one positive lymph node, ER positivity, PR positivity, and Her2 positivity were independent factors associated with better BCSS (**Table 2**). Multivariate Cox regression analysis identified nine independent predictors of OS and eight independent predictors of BCSS.

**Table 2:** Multivariate Cox analysis for OS and BCSS in the training cohort based on the results of LASSO regression.

Characteristic	OS		BCSS	
	aHR (95% CI)	P-value	aHR (95% CI)	P-value
Age (years)				
<40				
40-49	0.85 (0.60 - 1.20)	0.352	0.89 (0.62 - 1.27)	0.513
50-59	1.08 (0.78 - 1.49)	0.646	0.99 (0.70 - 1.40)	0.961
60-69	1.63 (1.19 - 2.25)	0.003	1.27 (0.89 - 1.80)	0.182
≥70	3.09 (2.25 - 4.23)	<0.001	1.89 (1.34 - 2.66)	<0.001
Marital status				
Married				
Others	1.45 (1.27 - 1.65)	<0.001	1.33 (1.12 - 1.58)	0.001
Grade				
I				
II	1.25 (1.01 - 1.54)	0.042	2.42 (1.59 - 3.67)	<0.001
III	1.78 (1.42 - 2.24)	<0.001	4.17 (2.73 - 6.36)	<0.001
T stage				
T1				
T2	1.63 (1.42 - 1.87)	<0.001	1.68 (1.39 - 2.02)	<0.001
Positive lymph nodes				
1				
2	1.05 (0.90 - 1.22)	0.540	1.10 (0.90 - 1.34)	0.355
3	1.64 (1.35 - 1.99)	<0.001	1.67 (1.31 - 2.13)	<0.001
LNR (%)				
≤15				
>15	1.20 (1.05 - 1.37)	0.007		
ER				
Negative				
Positive	0.73 (0.58 - 0.90)	0.004	0.73 (0.56 - 0.94)	0.017
PR				
Negative				
Positive	0.69 (0.58 - 0.83)	<0.001	0.59 (0.46 - 0.74)	<0.001
Her2				
Negative				
Positive			0.56 (0.44 - 0.72)	<0.001
Chemotherapy				
No				
Yes	0.54 (0.46 - 0.64)	<0.001		

OS, overall survival; BCSS, breast cancer-specific survival; LASSO, least absolute shrinkage and selection operator; aHR, adjusted hazard ratio; LNR, lymph node ratio; ER, estrogen receptor; PR, progesterone receptor; Her2, Human epidermal growth factor receptor 2.

### 3.3 Construction and validation of the nomograms

**Figure 3:**
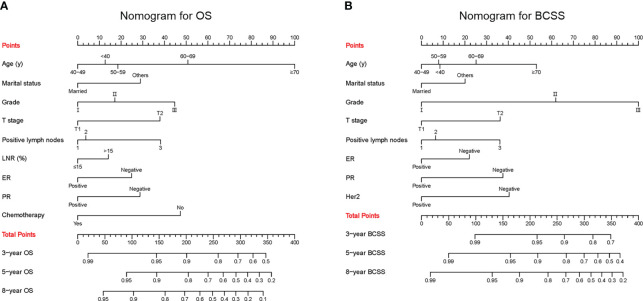
Predictive nomograms. **(A)** Nomogram for predicting 3-, 5- and 8-year OS. **(B)** Nomogram for predicting 3-, 5- and 8-year BCSS. LNR, lymph node ratio; ER, estrogen receptor; PR, progesterone receptor; Her2, Human epidermal growth factor receptor 2; OS, overall survival; BCSS, breast cancer-specific survival.

**Figure 4:**
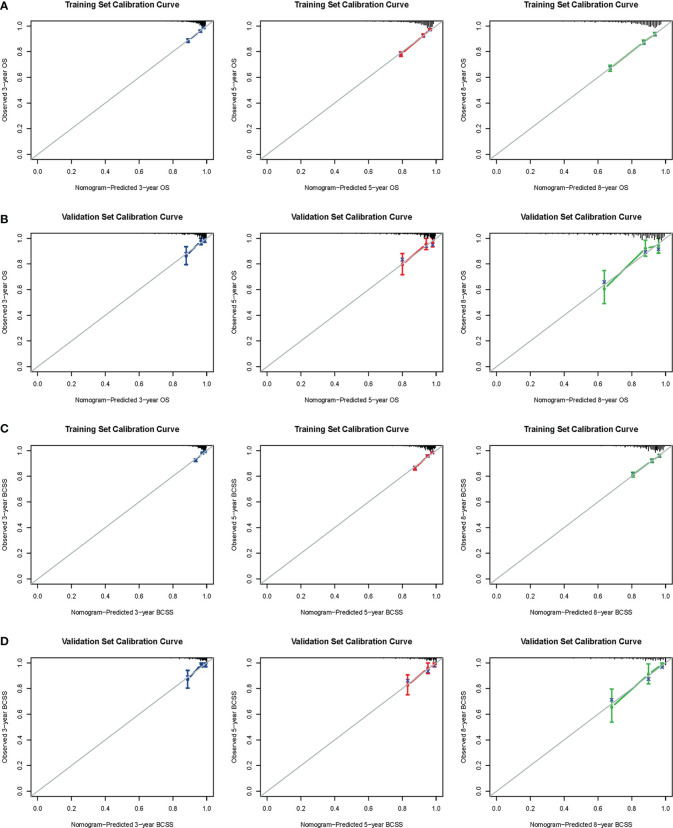
Calibration curves. 3-, 5-, and 8-year OS nomogram calibration curves for training **(A)** and validation cohorts **(B)**; 3-, 5-, and 8-year BCSS nomogram calibration curves for training **(C)** and validation cohorts **(D)**. OS, overall survival; BCSS, breast cancer-specific survival.

**Figure 5:**
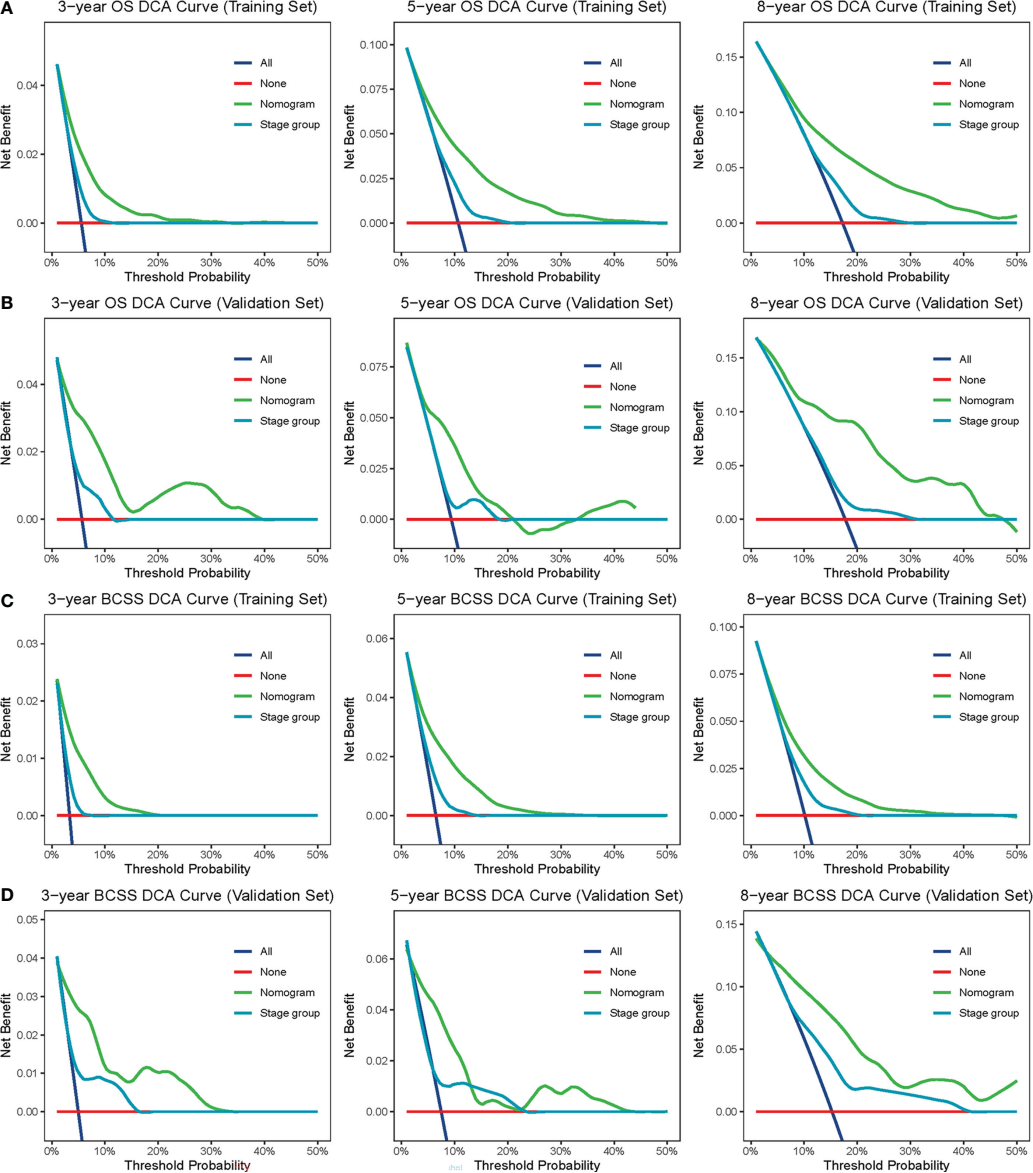
DCA curves. The DCA curves of nomogram and stage group (combination of the T stage and positive lymph nodes) in the training set **(A, B)** and validation set **(C, D)** were plotted based on 3-, 5-, and 8-year OS and BCSS, respectively. The green line represents nomogram, and the blue line represents stage group. DCA, decision curve analysis; OS, overall survival; BCSS, breast cancer-specific survival.

The confirmed factors were incorporated for constructing nomograms to predict 3-, 5-, and 8-year OS and BCSS (**Figure 3**). The C-indexes for the OS and BCSS nomograms were 0.734 (95% CI: 0.718–0.750) and 0.731 (95% CI: 0.661–0.803), respectively, in the training cohort and 0.733 (95% CI: 0.711–0.755) and 0.761 (95% CI: 0.683–0.840), respectively, in the validation cohort. The 3-, 5-, and 8-year calibration curves indicated excellent agreement between the nomogram-predicted and actual survival outcomes in the training and validation cohorts (**Figure 4**). Moreover, the 3-,5-, and 8-year DCA curves demonstrated that the net benefits of the nomograms were higher than that of stage grouping, which was defined by combining the T stage (T1 or T2) and the number of positive lymph nodes (one, two, or three) across nearly the entire range of threshold probabilities in both the training and validation cohorts (**Figure 5**).

### 3.4 Risk subgroup classification

A risk score was calculated for each patient using the nomograms, and the cut-off values were established using the X-tile software based on the survival data. Based on these risk scores, patients in the SEER and NSMC cohorts were categorized into different risk groups. For OS, patients were classified into low- (risk scores ≤ 131) and high- (risk scores ≥ 211) risk groups, while the remaining patients were classified into intermediate-risk groups. Similarly, for BCSS, patients were classified into low- (risk scores ≤ 164) and high- (risk scores ≥ 243) risk groups, with the remainder assigned to intermediate-risk groups (**Figure S1**). Furthermore, to assist the decision-making of PMRT more conveniently, we developed a public-accessible web calculator to predict the survival outcome and perform risk subgroup classification of T1-2N1 breast cancer (https://x5ve0t.shinyapps.io/Nomogram_for_predicting_OS_and_BCSS/). By selecting the nomogram-confirmed risk factors, the users of this calculator could receive the risk subgroup classification and survival outcomes.

**Figure 6:**
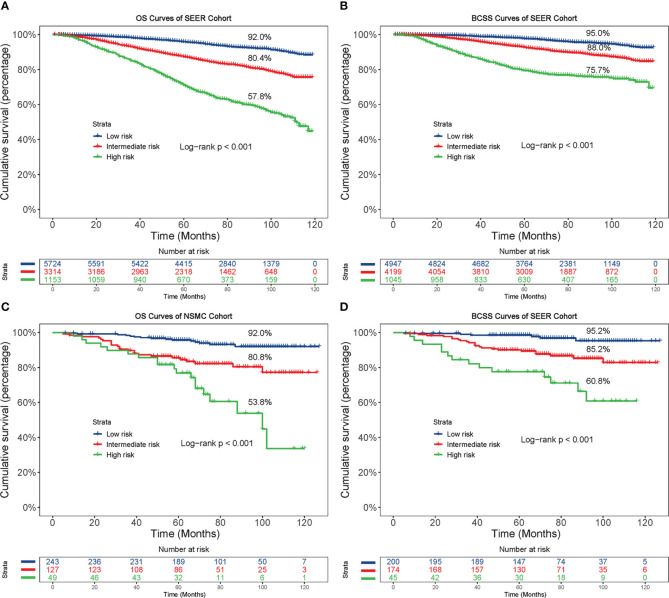
Kaplan-Meier curves based on the risk model. Based on the risk model, patients were divided into low-, intermediate- and high-risk groups, and then the OS and BCSS curves were plotted in the SEER cohort **(A, B)** and NSMC cohort **(C, D)**, respectively. OS, overall survival; BCSS, breast cancer-specific survival.

As depicted in **Figure 6**, the different risk groups had statistically significant differences in the survival outcomes. Briefly, in the low-risk groups, the 8-year OS and BCSS rates were 92.0% and 95.0%, respectively, in the SEER cohort and 92.0% and 95.2%, respectively, in the NSMC cohort. In the intermediate-risk groups, the 8-year OS and BCSS rates were 80.4% and 88.0%, respectively, in the SEER cohort and 80.8% and 85.2%, respectively, in the NSMC cohort. In the high-risk groups, the 8-year OS and BCSS rates were 57.8% and 75.7%, respectively, in the SEER cohort and 53.8% and 60.8%, respectively, in the NSMC cohort. The significant differences in survival between the three risk groups (p < 0.001) indicated the nomogram’s outstanding risk stratification ability.

### 3.5 Heterogeneity in the efficacy of PMRT across subgroups

**Figure 7:**
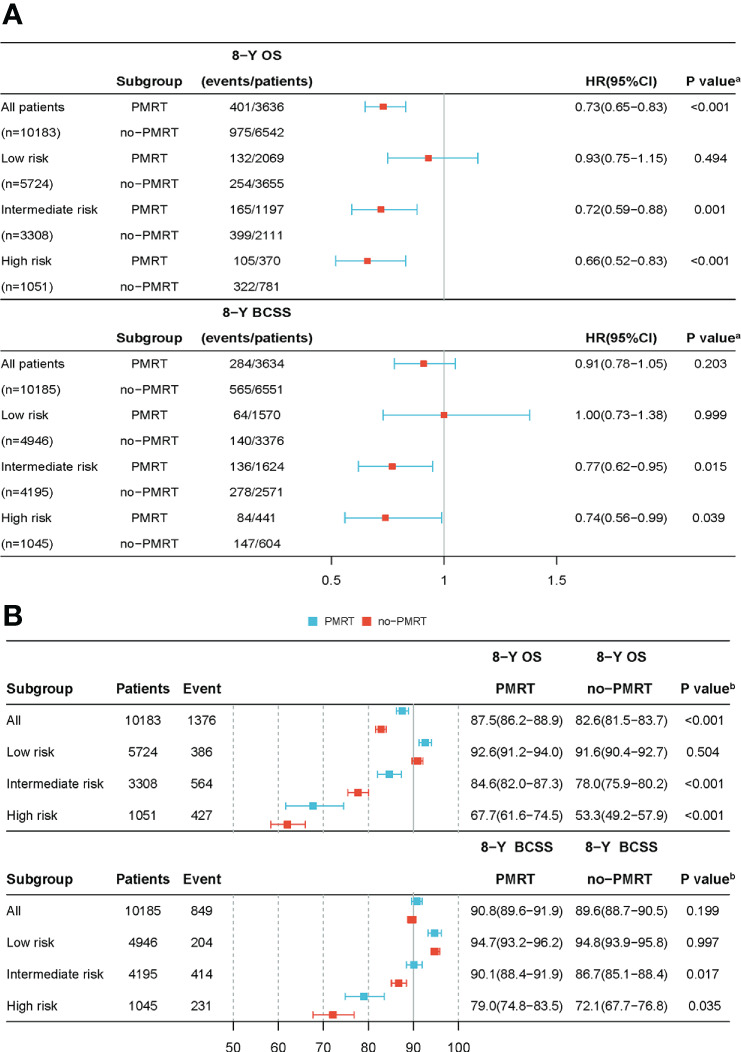
The forest plot of the IPTWs-adjusted cohort. The adjusted HR and 95% CI was calculated to estimate the effectiveness of PMRT use **(A)** in different risk groups after IPTWs. The adjusted Kaplan-Meier method was used to estimate OS and BCSS **(B)** rates between the PMRT and no-PMRT sets in various risk groups following IPTWs. **(A)** P value was calculated by Cox-regression; **(B)** P value was calculated by log-rank test. HR, hazard ratio; CI, confidence intervals; OS, overall survival; BCSS, breast cancer-specific survival; IPTWs, stabilized inverse probability of treatment weighting.

The standardized mean differences (SMDs) between most covariates were > 0.1, indicating an imbalance in the demographic and clinicopathological characteristics between the no-PMRT and PMRT groups in the three risk groups (**Tables S1**, **S2**). However, after adjustment by IPTWs, the SMDs for all variables were < 0.1 (**Tables 3**, **4**), indicating a balanced distribution of baseline characteristics between the no-PMRT and PMRT groups in the different risk groups. For all patients, PMRT was significantly associated with better OS (8-year OS: 87.6% vs. 82.6%, HR = 0.73, 95% CI: 0.65–0.83, P < 0.001) but not with better BCSS (8-year BCSS: 90.8% vs. 89.6%, HR = 0.91, 95% CI: 0.78–1.05, P = 0.203; **Figure 7**). Similar results were observed in the original cohort (**Figure S2**).

**Table 3:** The baseline characteristics of the patients in the IPTWs-adjusted cohort based on OS.

Characteristic	Low-risk			Intermediate-risk			High-risk		
	no-PMRT	PMRT	SMD	no-PMRT	PMRT	SMD	no-PMRT	PMRT	SMD
	(n=3655.4)	(n=2068.6)		(n=2110.9)	(n=1197.0)		(n=781.1)	(n=370.5)	
**Age (years)**			0.013			0.013			0.027
<40	457.2 (12.5)	255.9 (12.4)		162.4 (7.7)	93.2 (7.8)		3.4 (0.4)	1.6 (0.4)	
40-49	1354.4 (37.1)	770.9 (37.3)		229.9 (10.9)	130.3 (10.9)		4.9 (0.6)	2.3 (0.6)	
50-59	1265.6 (34.6)	707.4 (34.2)		478.0 (22.6)	272.6 (22.8)		13.4 (1.7)	6.2 (1.7)	
60-69	553.4 (15.1)	319.0 (15.4)		773.3 (36.6)	442.2 (36.9)		111.5 (14.3)	56.4 (15.2)	
≥70	24.9 (0.7)	15.3 (0.7)		467.2 (22.1)	258.8 (21.6)		647.9 (83.0)	304.0 (82.1)	
**Marital status**			0.010			0.016			0.015
Married	2734.8 (74.8)	1538.5 (74.4)		1095.1 (51.9)	630.4 (52.7)		206.0 (26.4)	95.3 (25.7)	
Others	920.7 (25.2)	530.1 (25.6)		1015.9 (48.1)	566.6 (47.3)		575.1 (73.6)	275.2 (74.3)	
**Grade**			0.009			0.011			0.027
I	618.9 (16.9)	343.4 (16.6)		199.5 (9.4)	110.1 (9.2)		59.0 (7.6)	30.7 (8.3)	
II	2037.1 (55.7)	1155.7 (55.9)		817.1 (38.7)	461.2 (38.5)		278.5 (35.7)	131.4 (35.5)	
III	999.5 (27.3)	569.4 (27.5)		1094.3 (51.8)	625.8 (52.3)		443.5 (56.8)	208.4 (56.3)	
**T stage**			0.002			0.005			0.001
T1	2029.6 (55.5)	1146.9 (55.4)		594.9 (28.2)	334.8 (28.0)		93.3 (11.9)	44.1 (11.9)	
T2	1625.8 (44.5)	921.7 (44.6)		1516.0 (71.8)	862.2 (72.0)		687.7 (88.1)	326.4 (88.1)	
**Positive lymph nodes**			0.002			0.013			0.017
1	2338.6 (64.0)	1322.2 (63.9)		1136.4 (53.8)	636.8 (53.2)		383.1 (49.1)	178.8 (48.2)	
2	1034.6 (28.3)	587.3 (28.4)		548.5 (26.0)	316.3 (26.4)		188.1 (24.1)	91.2 (24.6)	
3	282.3 (7.7)	159.1 (7.7)		426.0 (20.2)	243.9 (20.4)		209.8 (26.9)	100.5 (27.1)	
**LNR (%)**			0.001			0.014			0.017
≤15	2005.1 (54.9)	1135.5 (54.9)		953.5 (45.2)	532.6 (44.5)		290.3 (37.2)	134.7 (36.4)	
>15	1650.3 (45.1)	933.0 (45.1)		1157.4 (54.8)	664.5 (55.5)		490.8 (62.8)	235.8 (63.6)	
**ER**			0.004			0.009			0.012
Negative	204.2 (5.6)	117.2 (5.7)		560.4 (26.5)	322.4 (26.9)		182.6 (23.4)	88.5 (23.9)	
Positive	3451.3 (94.4)	1951.4 (94.3)		1550.5 (73.5)	874.7 (73.1)		598.4 (76.6)	282.0 (76.1)	
**PR**			0.002			0.015			0.037
Negative	464.6 (12.7)	264.4 (12.8)		814.1 (38.6)	470.3 (39.3)		328.6 (42.1)	162.7 (43.9)	
Positive	3190.8 (87.3)	1804.2 (87.2)		1296.8 (61.4)	726.7 (60.7)		452.4 (57.9)	207.8 (56.1)	
**Chemotherapy**			0.003			0.018			0.021
No	481.0 (13.2)	270.0 (13.1)		631.2 (29.9)	348.4 (29.1)		458.9 (58.7)	213.8 (57.7)	
Yes	3174.4 (86.8)	1798.6 (86.9)		1479.7 (70.1)	848.7 (70.9)		322.2 (41.3)	156.7 (42.3)	

OS, overall survival; IPTWs, stabilized inverse probability of treatment weighting; PMRT, postmastectomy radiotherapy; SMD, standardized mean difference; LNR, lymph node ratio; ER, estrogen receptor; PR, progesterone receptor.

**Table 4:** The baseline characteristics of the patients in the IPTWs-adjusted cohort based on BCSS.

Characteristic	Low-risk			Intermediate-risk			High-risk		
	no-PMRT	PMRT	SMD	no-PMRT	PMRT	SMD	no-PMRT	PMRT	SMD
	(n=3376.1)	(n=1569.9)		(n=2571.4)	(n=1623.8)		(n=603.7)	(n=440.8)	
**Age (years)**			0.013			0.011			0.018
<40	297.5 (8.8)	138.0 (8.8)		265.6 (10.3)	169.2 (10.4)		61.2 (10.1)	44.4 (10.1)	
40-49	1097.6 (32.5)	510.0 (32.5)		480.1 (18.7)	303.6 (18.7)		57.8 (9.6)	41.7 (9.5)	
50-59	1016.4 (30.1)	471.8 (30.1)		661.7 (25.7)	420.3 (25.9)		106.3 (17.6)	78.3 (17.8)	
60-69	700.5 (20.7)	331.6 (21.1)		617.4 (24.0)	392.8 (24.2)		120.6 (20.0)	90.9 (20.6)	
≥70	264.1 (7.8)	118.5 (7.6)		546.7 (21.3)	337.9 (20.8)		257.8 (42.7)	185.5 (42.1)	
**Marital status**			<0.001			0.006			0.009
Married	2493.7 (73.9)	1159.7 (73.9)		1381.2 (53.7)	877.3 (54.0)		235.6 (39.0)	170.1 (38.6)	
Others	882.4 (26.1)	410.2 (26.1)		1190.2 (46.3)	746.5 (46.0)		368.1 (61.0)	270.8 (61.4)	
**Grade**			0.003			0.007			0.006
I	913.8 (27.1)	422.8 (26.9)		20.2 (0.8)	13.6 (0.8)		0.0 (0.0)	0.0 (0.0)	
II	2006.1 (59.4)	934.0 (59.5)		1120.7 (43.6)	704.7 (43.4)		62.4 (10.3)	46.4 (10.5)	
III	456.3 (13.5)	213.1 (13.6)		1430.6 (55.6)	905.4 (55.8)		541.3 (89.7)	394.5 (89.5)	
**T stage**			0.006			0.012			0.002
T1	2081.6 (61.7)	963.7 (61.4)		681.3 (26.5)	421.4 (26.0)		45.5 (7.5)	33.4 (7.6)	
T2	1294.5 (38.3)	606.2 (38.6)		1890.1 (73.5)	1202.4 (74.0)		558.2 (92.5)	407.4 (92.4)	
**Positive lymph nodes**			0.003			0.009			0.009
1	2307.6 (68.3)	1071.0 (68.2)		1335.4 (51.9)	836.2 (51.5)		256.3 (42.5)	185.6 (42.1)	
2	863.1 (25.6)	402.8 (25.7)		746.9 (29.0)	477.5 (29.4)		160.9 (26.7)	119.0 (27.0)	
3	205.5 (6.1)	96.0 (6.1)		489.1 (19.0)	310.1 (19.1)		186.4 (30.9)	136.3 (30.9)	
**ER**			0.004			0.002			0.009
Negative	62.3 (1.8)	29.7 (1.9)		453.8 (17.6)	285.6 (17.6)		365.0 (60.5)	268.4 (60.9)	
Positive	3313.9 (98.2)	1540.1 (98.1)		2117.7 (82.4)	1338.2 (82.4)		238.7 (39.5)	172.4 (39.1)	
**PR**			0.011			0.002			0.01
Negative	192.3 (5.7)	93.3 (5.9)		841.1 (32.7)	532.8 (32.8)		484.3 (80.2)	355.4 (80.6)	
Positive	3183.9 (94.3)	1476.6 (94.1)		1730.3 (67.3)	1091.0 (67.2)		119.4 (19.8)	85.5 (19.4)	
**Her2**			0.012			<0.001			0.014
Negative	2765.6 (81.9)	1278.4 (81.4)		2145.5 (83.4)	1355.1 (83.5)		557.2 (92.3)	408.6 (92.7)	
Positive	610.6 (18.1)	291.4 (18.6)		425.9 (16.6)	268.7 (16.5)		46.5 (7.7)	32.3 (7.3)	

BCSS, breast cancer-specific survival; IPTWs, stabilized inverse probability of treatment weighting; PMRT, postmastectomy radiotherapy; SMD, standardized mean difference; LNR, lymph node ratio; ER, estrogen receptor; PR, progesterone receptor; Her2, Human epidermal growth factor receptor 2.

Subgroup analysis based on adjusted Cox regression showed significant heterogeneity in the effectiveness of PMRT on both OS and BCSS (**Figure 7**). In the IPTW-adjusted cohort, PMRT was significantly correlated with better OS in the intermediate- (8-year OS 84.6% vs. 78.0%, HR=0.72, 95% CI 0.59–0.88, P=0.001) and high- (8-year OS 67.7% vs. 53.3%, HR=0.66, 95% CI 0.52–0.83, P<0.001) risk groups, whereas it was not associated with OS in the low-risk group (8-year OS, 92.6% vs. 91.6%; HR=0.93, 95% CI 0.75–1.15, P=0.494). PMRT was also significantly correlated with better BCSS in the intermediate- (8-year BCSS 90.1% vs. 86.7%, HR=0.77, 95% CI 0.62–0.95, P=0.015) and high- (8-year BCSS 79.0% vs. 72.1%, HR=0.74, 95% CI 0.56–0.99, P=0.039) risk groups, while no association was seen with BCSS in the low-risk group (8-year BCSS: 94.7% vs. 94.8%, HR=1.00, 95% CI: 0.73–1.38, P = 0.999). The absolute survival benefit was more significant for patients in the high-risk group (8-year OS: 14.4%; 8-year BCSS: 6.9%) than the intermediate-risk group (8-year OS: 6.6%; 8-year BCSS: 3.4%). These findings were consistent with those from the original cohort (**Figure S2**).

## 4 Discussion

By reviewing the clinicopathological characteristics of patients with T1-2N1 breast cancer in the SEER database, we constructed nomograms to predict the OS and BCSS of patients with T1-2N1 breast cancer and validated them in the Chinese population. The C-index and calibration curves demonstrated the nomograms’ excellent discrimination and accuracy. The DCA results indicated that the nomograms outperformed the combination of tumor size and the number of positive lymph nodes. In addition, we developed a new risk stratification system to estimate the effect of PMRT on the different risk groups. Interestingly, we found that the low-risk subgroups of patients appeared to gain no survival benefit from PMRT. The high-risk group obtained a greater absolute benefit from PMRT than the intermediate-risk group did.

In the late 1990s, the DBCG 82 b&c and Columbia trials successively demonstrated that PMRT improved the OS in patients with 1 to 3 positive lymph nodes (10–12). This finding was later reinforced by a meta-analysis conducted in 2014 by the Early Breast Cancer Trialists Collaborative Group. The analysis showed that PMRT significantly reduced this patient population’s 20-year breast cancer mortality (13). However, the generalizability of these data was questioned for the outdated therapy the patients received (14–19). In current clinical practice, advances in diagnostic and surgical techniques, as well as systemic treatment, have resulted in a limited survival benefit of PMRT within certain subsets of patients. Therefore, new studies are warranted to evaluate the efficacy of modern PMRT in this patient population.

Recent studies have identified some adverse prognostic factors associated with survival in patients with T1-2N1 breast cancer, including young age, greater lymph node disease burden, large tumor size, high histological grade, lymphovascular invasion, and negative hormone receptors (3, 7, 14, 15, 17, 20–27). However, the weights and proportional influences of these prognostic factors were not specified. Several researchers have attempted to use combinations of prognostic factors, such as tumor size and the number of positive lymph nodes, to define subgroups with more specific risks than single factors alone (3, 28). However, the information represented by such combinations is limited because other critical prognostic factors need to be considered. Furthermore, our nomograms presented better clinical applicability than the combination of tumor size and the number of positive lymph nodes. Therefore, the nomograms we developed incorporated all independent prognostic predictors, which provided a more effective individualized survival prediction tool for this population to assist in PMRT decision-making.

Typically, young age is regarded as a risk factor for developing aggressive breast cancer. Several retrospective series have found that young patients with T1-2N1 breast cancer have a worse prognosis (5, 20, 26, 27). However, our analysis demonstrated that patients aged ≥70 years old had worse OS and BCSS, and this finding was similar to that of several previous studies (4, 29–31). Hence, the influence of age on the survival of patients with T1-2N1 breast cancer remains controversial, and this observation cannot be explained solely by the higher all-cause mortality rate in patients over 70 years of age.

A growing amount of research has demonstrated that being married is an independent protective factor for survival in patients with breast cancer (32–36). Married patients are able to receive more mental and financial support and demonstrate better compliance. Similar findings have recently been reported in patients with T1-2N1 breast cancer (4). Given the current research situation, we included marital status in the models to explore the relationship between marital status and survival. The findings demonstrated that married patients obtained better survival outcomes than other (unmarried/separated/divorced/widowed) patients, and incorporating marital status improved the stability and robustness of the model.

Due to the lack of high-level prospective research evidence, a consensus has not yet been reached regarding the indications for PMRT in patients with T1-2N1M0. The ongoing SUPREMO trial (www.supremo-trial.com) recruited 1688 women with breast cancer (including pT1-2N1M0 breast cancer) (37), the results of which are expected to help clarify this issue.

With the gradual adoption of commercial polygenic assays, clinicians are considering the potential of genomic assays to guide decisions regarding adjuvant radiotherapy. Among these, the 21-gene recurrence score (RS) and 28-gene recurrence index (RI) have generated substantial debate (38–42). At the 17th International Breast Cancer Conference in St. Gallen in 2021, the panel concluded that genomic signatures still could not be used to help decide on using PMRT (89%) or omitting it (84%) (43). In other words, the use of polygenic assays to guide the use of PMRT currently lacks the support of high-level evidence. The TAILOR RT trial (NCT03488693) is currently underway (44). The publication of its final trial data is anticipated to clarify the predictive significance of RS for PMRT. In conclusion, polygenic assays are expected to provide a new direction for precisely individualized radiation and chemotherapy for breast cancer.

Our study had several limitations. Firstly, the lack of detailed information regarding chemotherapy, endocrine treatment, and radiation regimens in the SEER database prevented us from conducting a more specific analysis. Secondly, we could not obtain data from the database of patients with local recurrence and distant metastases to determine the specific function of PMRT. Thirdly, some bias is inevitable due to the retrospective nature of this study. Finally, the role of PMRT after neoadjuvant chemotherapy is currently under active investigation, and ongoing randomized trials (National Surgical Adjuvant Breast and Bowel Project B-51 and Alliance 011202) might help clarify this subject (45, 46).

## 5 Conclusion

The nomograms we constructed could accurately identify low-risk patients with better prognoses who could not benefit from PMRT. Compared with the intermediate-risk group, the absolute and relative survival benefit was more significant in the high-risk group. Prospective validation of the conclusions in the present study is recommended in a larger cohort. Some of the current ongoing clinical trials may provide some reference for the best decision-making regarding PMRT for patients with T1-2N1M breast cancer.

## Data availability statement

Publicly available datasets were analyzed in this study. This data can be found here: https://seer.cancer.gov/.

## Ethics statement

The studies involving human participants were reviewed and approved by the Ethics Committee of the Affiliated Hospital of North Sichuan Medical College Number: 2022ER055-1. Written informed consent from the participants’ legal guardian/next of kin was not required to participate in this study in accordance with the national legislation and the institutional requirements.

## Author contributions

YG, LH, and XZ designed the study. HP and YL wrote the main manuscript. LZ and XL extracted the data from the SEER database. FL collected data for external validation. JC, SQ, and YT performed the statistical analysis. All authors contributed to the article and approved the submitted version.
