# Supplementary material for: Development and validation of nomograms for predicting survival outcomes in patients with T1-2N1 breast cancer to identify those who could not benefit from postmastectomy radiotherapy
Source: Front Oncol. 2023 Mar 28;13:1112687. doi: 10.3389/fonc.2023.1112687 (PMC10086367; doi:10.3389/fonc.2023.1112687)
Supplement: Supplementary file 1 [file DataSheet_1.docx]

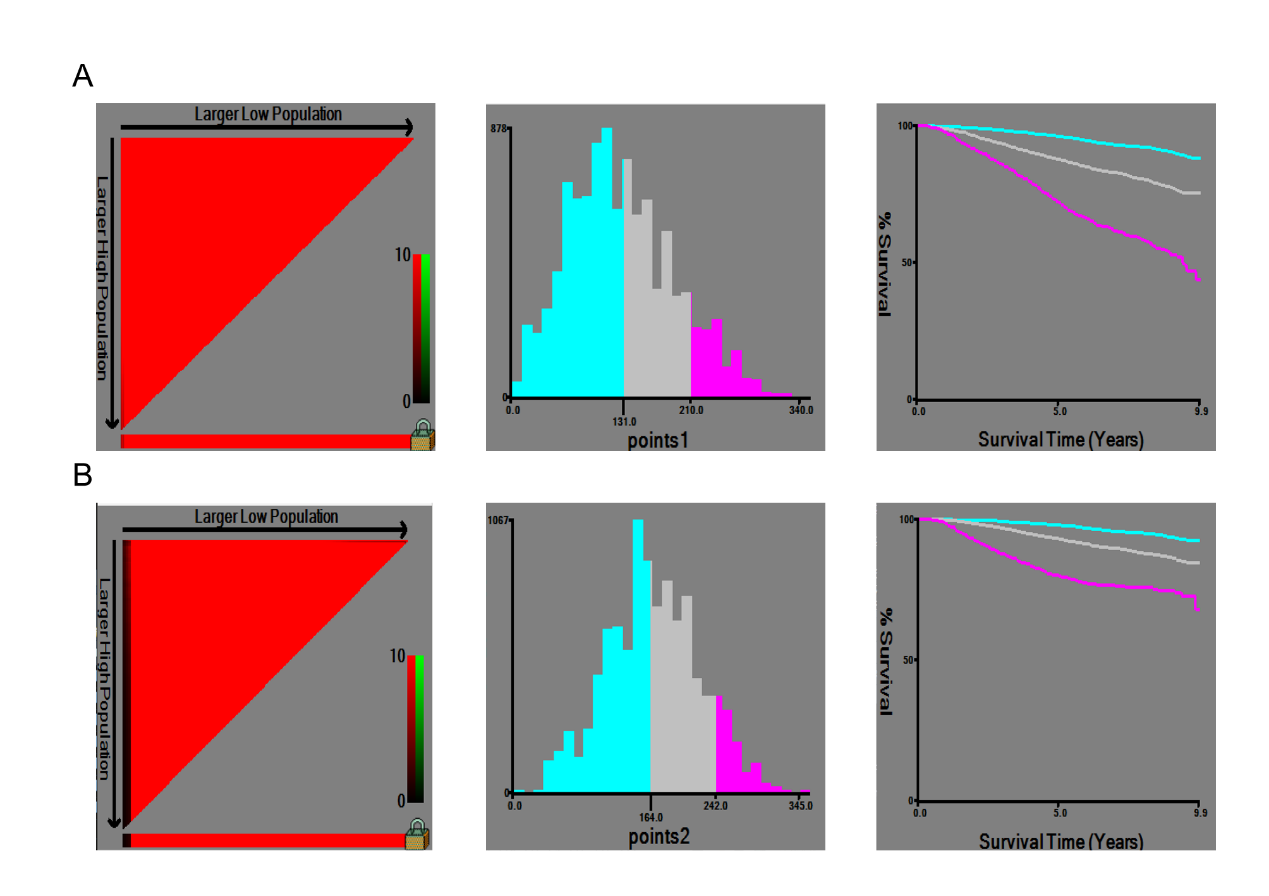


**FIGURE S1: X-tile analysis of survival data.**

X-tile plots of the SEER cohort for OS and BCSS is displayed in the (A) and (B), respectively. OS, overall survival; BCSS, breast cancer-specific survival.


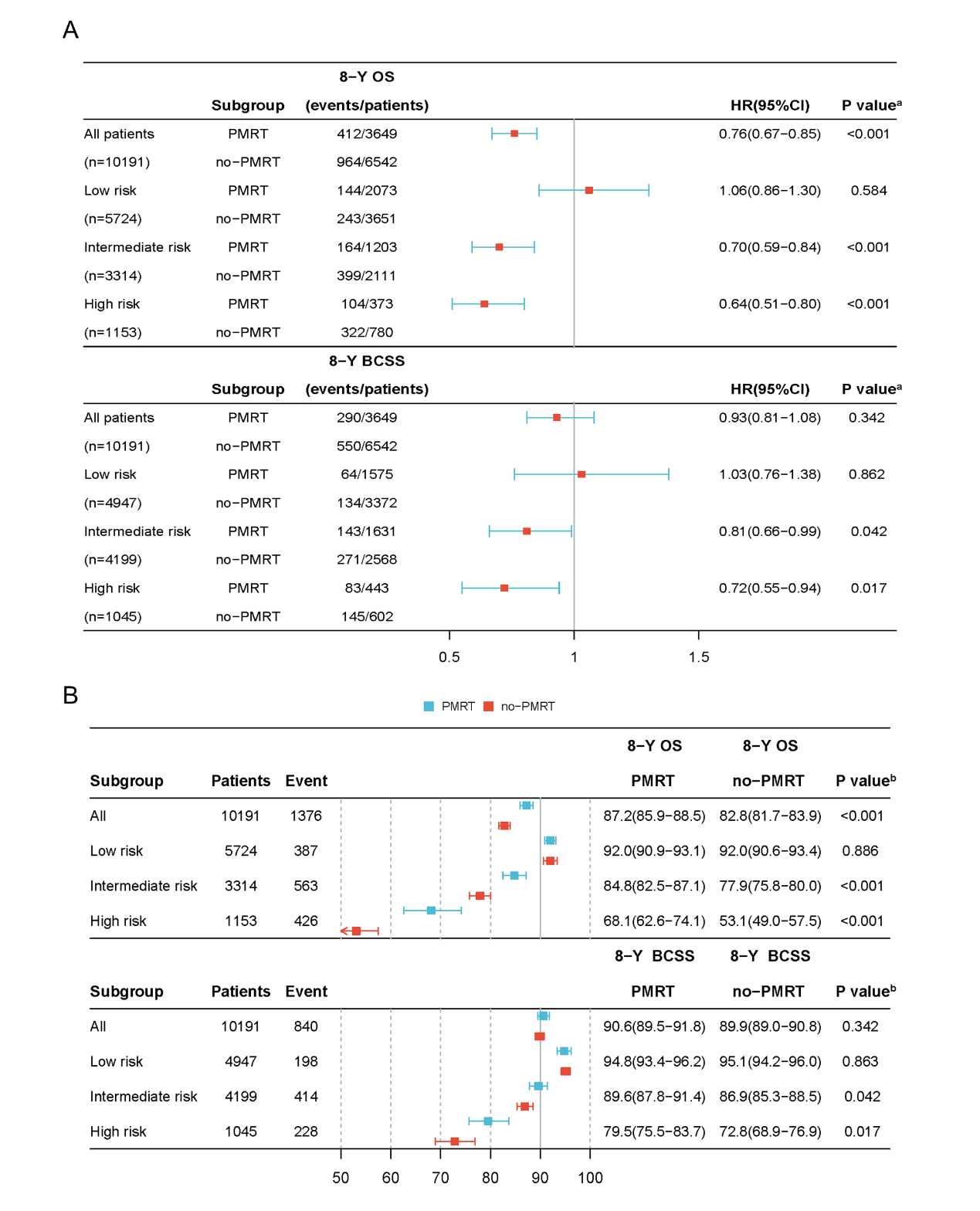


**FIGURE S2: The forest plot of the original cohort.**

The HR and 95 % CI was calculated to estimate the effectiveness of PMRT use in different risk groups before IPTWs (A). The Kaplan-Meier method were used to estimate OS and BCSS rates between the PMRT and no-PMRT sets in various risk groups before IPTWs (B). a. P value was calculated by Cox-regression; b. P value was calculated by log-rank test. HR, hazard ratio; CI; confidence intervals; OS, overall survival; BCSS, breast cancer-specific survival; IPTWs, stabilized inverse probability of treatment weighting.

**TABLE S1: The baseline characteristics of the patients in the original cohort based on OS.**

| **Characteristic** | **Low-risk** | | |  | **Intermediate-risk** | | |  | **High-risk** | | |
| --- | --- | --- | --- | --- | --- | --- | --- | --- | --- | --- | --- |
|  | **no-PMRT** | **PMRT** | **SMD** |  | **no-PMRT** | **PMRT** | **SMD** |  | **no-PMRT** | **PMRT** | **SMD** |
|  | (n=3651) | (n=2073) |  |  | (n=2111) | (n=1203) |  |  | (n=780) | (n=373) |  |
| **Age (years)** |  |  | 0.217 |  |  |  | 0.433 |  |  |  | 0.350 |
| <40 | 370 (10.1) | 331 (16.0) |  |  | 111 (5.3) | 145 (12.1) |  |  | 2 (0.3) | 3 (0.8) |  |
| 40-49 | 1332 (36.5) | 798 (38.5) |  |  | 183 (8.7) | 179 (14.9) |  |  | 1 (0.1) | 6 (1.6) |  |
| 50-59 | 1322 (36.2) | 667 (32.2) |  |  | 449 (21.3) | 304 (25.3) |  |  | 12 (1.5) | 8 (2.1) |  |
| 60-69 | 593 (16.2) | 272 (13.1) |  |  | 803 (38.0) | 408 (33.9) |  |  | 82 (10.5) | 77 (20.6) |  |
| ≥70 | 34 (0.9) | 5 (0.2) |  |  | 565 (26.8) | 167 (13.9) |  |  | 683 (87.6) | 279 (74.8) |  |
| **Marital status** |  |  | 0.019 |  |  |  | 0.087 |  |  |  | 0.104 |
| Married | 2724 (74.6) | 1564 (75.4) |  |  | 1061 (50.3) | 657 (54.6) |  |  | 196 (25.1) | 111 (29.8) |  |
| Others | 927 (25.4) | 509 (24.6) |  |  | 1050 (49.7) | 546 (45.4) |  |  | 584 (74.9) | 262 (70.2) |  |
| **Grade** |  |  | 0.157 |  |  |  | 0.303 |  |  |  | 0.102 |
| Ⅰ | 692 (19.0) | 284 (13.7) |  |  | 241 (11.4) | 71 (5.9) |  |  | 65 (8.3) | 22 (5.9) |  |
| Ⅱ | 2017 (55.2) | 1158 (55.9) |  |  | 876 (41.5) | 398 (33.1) |  |  | 282 (36.2) | 132 (35.4) |  |
| Ⅲ | 942 (25.8) | 631 (30.4) |  |  | 994 (47.1) | 734 (61.0) |  |  | 433 (55.5) | 219 (58.7) |  |
| **T stage** |  |  | 0.216 |  |  |  | 0.319 |  |  |  | 0.143 |
| T1 | 2176 (59.6) | 1014 (48.9) |  |  | 702 (33.3) | 233 (19.4) |  |  | 104 (13.3) | 33 (8.8) |  |
| T2 | 1475 (40.4) | 1059 (51.1) |  |  | 1409 (66.7) | 970 (80.6) |  |  | 676 (86.7) | 340 (91.2) |  |
| **Positive lymph nodes** |  |  | 0.410 |  |  |  | 0.528 |  |  |  | 0.581 |
| 1 | 2580 (70.7) | 1093 (52.7) |  |  | 1316 (62.3) | 469 (39.0) |  |  | 447 (57.3) | 120 (32.2) |  |
| 2 | 900 (24.7) | 712 (34.3) |  |  | 504 (23.9) | 352 (29.3) |  |  | 182 (23.3) | 96 (25.7) |  |
| 3 | 171 (4.7) | 268 (12.9) |  |  | 291 (13.8) | 382 (31.8) |  |  | 151 (19.4) | 157 (42.1) |  |
| **LNR (%)** |  |  | 0.231 |  |  |  | 0.285 |  |  |  | 0.399 |
| ≤15 | 2153 (59.0) | 985 (47.5) |  |  | 1062 (50.3) | 437 (36.3) |  |  | 337 (43.2) | 92 (24.7) |  |
| >15 | 1498 (41.0) | 1088 (52.5) |  |  | 1049 (49.7) | 766 (63.7) |  |  | 443 (56.8) | 281 (75.3) |  |
| **ER** |  |  | 0.014 |  |  |  | 0.104 |  |  |  | 0.105 |
| Negative | 207 (5.7) | 111 (5.4) |  |  | 525 (24.9) | 355 (29.5) |  |  | 170 (21.8) | 98 (26.3) |  |
| Positive | 3444 (94.3) | 1962 (94.6) |  |  | 1586 (75.1) | 848 (70.5) |  |  | 610 (78.2) | 275 (73.7) |  |
| **PR** |  |  | 0.019 |  |  |  | 0.168 |  |  |  | 0.054 |
| Negative | 454 (12.4) | 271 (13.1) |  |  | 754 (35.7) | 528 (43.9) |  |  | 318 (40.8) | 162 (43.4) |  |
| Positive | 3197 (87.6) | 1802 (86.9) |  |  | 1357 (64.3) | 675 (56.1) |  |  | 462 (59.2) | 211 (56.6) |  |
| **Chemotherapy** |  |  | 0.332 |  |  |  | 0.442 |  |  |  | 0.261 |
| No | 621 (17.0) | 134 (6.5) |  |  | 780 (36.9) | 213 (17.7) |  |  | 493 (63.2) | 188 (50.4) |  |
| Yes | 3030 (83.0) | 1939 (93.5) |  |  | 1331 (63.1) | 990 (82.3) |  |  | 287 (36.8) | 185 (49.6) |  |

OS, overall survival; IPTWs, stabilized inverse probability of treatment weighting; PMRT, postmastectomy radiotherapy; SMD, standardized mean difference; LNR, lymph node ratio; ER, estrogen receptor; PR, progesterone receptor.

**TABLE S2: The baseline characteristics of the patients in the original cohort based on BCSS.**

| **Characteristic** | **Low-risk** | | |  | **Intermediate-risk** | | |  | **High-risk** | | |
| --- | --- | --- | --- | --- | --- | --- | --- | --- | --- | --- | --- |
|  | **no-PMRT** | **PMRT** | **SMD** |  | **no-PMRT** | **PMRT** | **SMD** |  | **no-PMRT** | **PMRT** | **SMD** |
|  | (n=3372) | (n=1575) |  |  | (n=2568) | (n=1631) |  |  | (n=602) | (n=443) |  |
| **Age (years)** |  |  | 0.282 |  |  |  | 0.364 |  |  |  | 0.387 |
| <40 | 225 (6.7) | 203 (12.9) |  |  | 213 (8.3) | 216 (13.2) |  |  | 45 (7.5) | 60 (13.5) |  |
| 40-49 | 1061 (31.5) | 556 (35.3) |  |  | 417 (16.2) | 367 (22.5) |  |  | 38 (6.3) | 60 (13.5) |  |
| 50-59 | 1034 (30.7) | 456 (29.0) |  |  | 638 (24.8) | 446 (27.3) |  |  | 111 (18.4) | 77 (17.4) |  |
| 60-69 | 742 (22.0) | 281 (17.8) |  |  | 624 (24.3) | 382 (23.4) |  |  | 112 (18.6) | 94 (21.2) |  |
| ≥70 | 310 (9.2) | 79 (5.0) |  |  | 676 (26.3) | 220 (13.5) |  |  | 296 (49.2) | 152 (34.3) |  |
| **Marital status** |  |  | 0.075 |  |  |  | 0.127 |  |  |  | 0.191 |
| Married | 2457 (72.9) | 1199 (76.1) |  |  | 1315 (51.2) | 938 (57.5) |  |  | 209 (34.7) | 195 (44.0) |  |
| Others | 915 (27.1) | 376 (23.9) |  |  | 1253 (48.8) | 693 (42.5) |  |  | 393 (65.3) | 248 (56.0) |  |
| **Grade** |  |  | 0.141 |  |  |  | 0.06 |  |  |  | 0.031 |
| Ⅰ | 975 (28.9) | 367 (23.3) |  |  | 23 (0.9) | 10 (0.6) |  |  | 0 (0.0) | 0 (0.0) |  |
| Ⅱ | 1976 (58.6) | 963 (61.1) |  |  | 1135 (44.2) | 682 (41.8) |  |  | 64 (10.6) | 43 (9.7) |  |
| Ⅲ | 421 (12.5) | 245 (15.6) |  |  | 1410 (54.9) | 939 (57.6) |  |  | 538 (89.4) | 400 (90.3) |  |
| **T stage** |  |  | 0.172 |  |  |  | 0.173 |  |  |  | 0.022 |
| T1 | 2177 (64.6) | 885 (56.2) |  |  | 761 (29.6) | 360 (22.1) |  |  | 44 (7.3) | 35 (7.9) |  |
| T2 | 1195 (35.4) | 690 (43.8) |  |  | 1807 (70.4) | 1271 (77.9) |  |  | 558 (92.7) | 408 (92.1) |  |
| **Positive lymph nodes** |  |  | 0.402 |  |  |  | 0.443 |  |  |  | 0.478 |
| 1 | 2506 (74.3) | 884 (56.1) |  |  | 1528 (59.5) | 661 (40.5) |  |  | 309 (51.3) | 137 (30.9) |  |
| 2 | 726 (21.5) | 529 (33.6) |  |  | 702 (27.3) | 509 (31.2) |  |  | 158 (26.2) | 122 (27.5) |  |
| 3 | 140 (4.2) | 162 (10.3) |  |  | 338 (13.2) | 461 (28.3) |  |  | 135 (22.4) | 184 (41.5) |  |
| **ER** |  |  | 0.023 |  |  |  | 0.074 |  |  |  | 0.027 |
| Negative | 58 (1.7) | 32 (2.0) |  |  | 485 (18.9) | 262 (16.1) |  |  | 359 (59.6) | 270 (60.9) |  |
| Positive | 3314 (98.3) | 1543 (98.0) |  |  | 2083 (81.1) | 1369 (83.9) |  |  | 243 (40.4) | 173 (39.1) |  |
| **PR** |  |  | 0.002 |  |  |  | 0.043 |  |  |  | 0.041 |
| Negative | 189 (5.6) | 89 (5.7) |  |  | 859 (33.5) | 513 (31.5) |  |  | 478 (79.4) | 359 (81.0) |  |
| Positive | 3183 (94.4) | 1486 (94.3) |  |  | 1709 (66.5) | 1118 (68.5) |  |  | 124 (20.6) | 84 (19.0) |  |
| **Her2** |  |  | 0.086 |  |  |  | 0.016 |  |  |  | 0.186 |
| Negative | 2808 (83.3) | 1259 (79.9) |  |  | 2134 (83.1) | 1365 (83.7) |  |  | 543 (90.2) | 421 (95.0) |  |
| Positive | 564 (16.7) | 316 (20.1) |  |  | 434 (16.9) | 266 (16.3) |  |  | 59 (9.8) | 22 (5.0) |  |

BCSS, breast cancer-specific survival; IPTWs, stabilized inverse probability of treatment weighting; PMRT, postmastectomy radiotherapy; SMD, standardized mean difference; LNR, lymph node ratio; ER, estrogen receptor; PR, progesterone receptor; Her2, Human epidermal growth factor receptor 2.
